# Impact of risk factors on functional status in maintenance hemodialysis patients

**DOI:** 10.1186/s40001-017-0298-1

**Published:** 2017-12-28

**Authors:** Jin-Bor Chen, Wen-Chin Lee, Ben-Chung Cheng, Sin-Hua Moi, Cheng-Hong Yang, Yu-Da Lin

**Affiliations:** 1 Division of Nephrology, Department of Internal Medicine, Kaohsiung Chang Gung Memorial Hospital and Chang Gung University College of Medicine, Kaohsiung, 83301 Taiwan; 2 Department of Electronic Engineering, National Kaohsiung University of Applied Sciences, Kaohsiung, 80778 Taiwan; 3 Graduate Institute of Clinical Medicine, Kaohsiung Medical University, Kaohsiung, 80708 Taiwan

**Keywords:** Hemodialysis, Functional status, Karnofsky Performance Status, Classification and regression tree approach

## Abstract

**Objectives:**

To survey by measuring patient’s functional status which is crucial when end-stage renal disease patients begin a dialysis program. The influence of the disease on patients can be examined by the measurement of Karnofsky Performance Status (KPS) scores, together with a quality of life survey, and clinical variables.

**Methods:**

The details for the dataset in the study were collected from patients receiving regular hemodialysis (HD) in one hospital, which were available retrospectively for 1166 patients during the 5-year study period. KPS scores were applied for quantifying functional status. To identify risk factors for functional status, clinical factors including demographics, laboratory data, and HD vintage were selected. This study applied a classification and regression tree approach (CART) and logistic regression to determine risk factors on functional impairment among HD patients.

**Results:**

Ten risk factors were identified by CART and regression model (age, primary kidney disease subclass, treatment years, hemoglobin, albumin, creatinine, phosphorus, intact parathyroid hormone, ferritin, and cardiothoracic ratio). The results of logistic regression with selected interaction models showed older age or higher hematocrit, blood urea nitrogen, and glucose levels could significantly increase the log-odds of obtaining low KPS scores at in-person visits.

**Conclusions:**

In interaction results, the combination of older age with higher albumin level and higher creatinine level with longer HD treatment years could significantly decrease the log-odds of a low KPS score assessment during in-person visits. Age, hemoglobin, albumin, urea, creatinine levels, primary kidney disease subclass, and HD duration are the major determinants for functional status in HD patients.

**Electronic supplementary material:**

The online version of this article (10.1186/s40001-017-0298-1) contains supplementary material, which is available to authorized users.

## Background

Dialysis therapy is the mainstay of treatment for end-stage renal disease (ESRD). In recent years, equal importance has been placed not only on dialysis adequacy, but also on quality of life (QoL) in order to reduce mortality in dialysis patients [1–6]. In addition, a functionally impaired state in ESRD patients at the start of dialysis therapy also contributes to unfavorable outcomes. A previous study reported that poor functional status scores were associated with mortality after a 3-year follow-up in hemodialysis (HD) patients [7]. Moreover, the study also revealed functional status scores and QoL scores were independently correlated with the risk of mortality in new dialysis patients [8]. A recent study retrospectively examined the prognostic significance of physical activity changes by conducting a 7-year follow-up on patients receiving HD. The results demonstrated that reductions in physical activity were significantly associated with poor prognoses independent of baseline physical activity [9]. Physical inactivity is a component of frailty. Frailty scores were reported as year-to-year variability in patients receiving HD. Markers of inflammation and hospitalization were independently reported as being associated with worsening frailty [10]. Thus, a survey of the patient’s functional status using a simple measure is crucial when ESRD patients begin a dialysis program.

The most common measure for functional status assessment is the Karnofsky Performance Status (KPS) scale. The measurement of KPS scores must be performed by trained observers to maintain reliability. A prior study demonstrated that KPS measurements can be a useful tool when used by trained observers [11]. KPS has been widely used for quantifying the functional status of cancer patients, and a poor KPS score predicts poor survival in cancer patients [11, 12]. Owing to the noted pitfalls in the original KPS scale, a modified version of the KPS was proposed in order to study the functional status of dialysis patients [7, 13]. It consists of 14 different levels of activity, ranging from < 30 (hospitalized, progressive disability process) to ≥ 96 (normal function). According to the modified assessment values of the KPS, a lower KPS score has been demonstrated to be associated with early mortality in HD patients [7]. In addition, KPS scores have shown a significant positive correlation with the domains of the Short Form (SF)-36 health survey in HD patients [14]. Based on the aforementioned background, a KPS measurement is frequently used to complement medical information, together with a QoL survey, and clinical variables to examine the influence of the disease on patients.

In the present study, we used the classification and regression tree approach (CART) approach to identify the risk factors related to functional status in HD patients. In addition, we compared the results yielded by the CART algorithm to those produced by a logistic regression model to obtain complementary evidence in the identification of risk factors for functional impairment in HD patients.

## Methods

### Data sources

For this retrospective cohort study, patients who received maintenance outpatient HD at Kaohsiung Chang Gung Memorial Hospital in Taiwan were enrolled. The follow-up period was from January 1, 2009, to December 31, 2013. Patients for whom data were incomplete and those lost to follow-up during the study period were excluded. A total of 1166 patients were eligible for inclusion in the KPS analysis. The protocol for the study was approved by the Committee on Human Research at the Kaohsiung Chang Gung Memorial Hospital (101-1595B) for data review, and was conducted in accordance with the principles of the Declaration of Helsinki.

### Baseline measurements

Laboratory values for the blood analysis and dialysis parameters included the intact parathyroid hormone (iPTH), urea-Daugirdas estimation of fractional removal of urea per dialysis treatment (Kt/V) [3], and normalized protein catabolic rate (nPCR), all of which were measured at the initial rate after enrolment. The cardiothoracic ratio (CT ratio) was assessed upon completion of the first year after enrollment. Initial serum calcium (Ca) was corrected using the following equation for serum albumin level < 4.0 g/dL: measured total Ca (mg/dL) + 0.8 × [4.0 − serum albumin (g/dL)].

Modified KPS scores were recorded once yearly by trained nurses at the HD unit. KPS scores comprised 14 different levels of activity, ranging from < 30 (hospitalized, progressive fatal process) to ≥ 96 (normal function) [7]. A low KPS score was defined as KPS < 80 and a high score as KPS ≥ 80.

### In-person visit measurements

To adhere to the in-person visit analysis design, laboratory values for blood analysis were measured monthly during the study period; exceptions were ferritin, which was measured every 3 months, and iPTH, Kt/V, and the nPCR, all of which were measured every 6 months. The CT ratio was assessed yearly. The KPS scores used were measured at each in-person visit between 2009 and 2013.

### CART analysis

The CART is a binary recursive partitioning algorithm that represents a model-free and nonparametric method for exploring nonlinear associations with a low false-positive rate [15]. The CART algorithm has been successfully applied to a variety of biological studies, for example, survival analysis [16], gene-environment interactions [17, 18], and prognostic models of mortality [19]. The CART algorithm was implemented using Salford Predictive Modeler software (version 7.0). In the CART setting, tree splitting was performed until the terminal nodes reached a prespecified minimum size of 10 subjects. The optimal tree structure was evaluated by the one standard error (1-SE) rule and tenfold cross validation (CV). Subgroups of individuals with differential risk patterns were detected in the different orders of nodes, indicating the presence of associations of assigned variables.

### Statistical analysis

The baseline characteristics were calculated using descriptive statistics (means, standard deviation [SD]), and percentages. The differences between both groups were evaluated by the *χ* ^2^ test, Fisher’s exact test, and independent two-sample *t* test. Clinical features of the study population were used to predict the KPS score at each in-person visit using the multivariate logistic regression model without interactions. The linked nodes from the decision tree were selected as interaction items to predict the KPS score level at each in-person visit using the logistic regression model with interactions estimation. AIC (Akaike Information Criterion) values were estimated for logistic regression models with and without interactions; the logistic model with a two-order interaction provided a better AIC value. The 95% confidence interval (95% CI) and a *P* value were used to determine statistical significance. All statistical analyses were performed using STATA software (version 11.1).

## Results

### Participant characteristics

A total of 1166 HD patients were enrolled. The mean age was 60.87 years. The mean HD duration was 7.87 years. The HD frequency approached thrice weekly and each HD time approached 4 h. Males and females were found with near-equal distribution. The majority was nondiabetic. Systemic diseases (diabetes mellitus [DM], systemic lupus erythematous, gout, liver cirrhosis, cardiac failure, etc.) (Additional file 1: Table S1) were the leading causes of kidney failure in HD patients. There were 143 deaths (12.26%) during the follow-up period (Table 1).

**Table 1:** Baseline characteristics of the study population (*N* = 1166)

Variables	Total	
	*N*	SD
Age (mean, SD) (years)	60.87	12.05
Treatment of year (mean, SD)	7.87	5.78
The number of HD a week (mean, SD)	2.93	0.27
Each dialysis time (mean, SD) (hours)	3.91	0.24
Dialyzer surface area (m^2^)	2.16	0.25
Gender (*n*, %)		
Male	572	49.06
Female	594	50.94
DM (*n*, %)		
No	797	68.35
Yes	369	31.65
Primary disease subclass (*n*, %)		
Glomerulonephritis	279	23.93
Systemic diseases	641	54.97
Obstructive nephropathy	14	1.20
Hemolytic uremic syndrome	2	0.17
Polycystic kidney disease	16	1.37
Others	51	4.37
Unknown cause	162	13.89
Use of vitamin D (*n*, %)		
No	708	60.72
Yes	458	39.28
Use of antihypertensive (*n*, %)		
No	573	49.14
Yes	593	50.86
Use of iron (*n*, %)		
No	881	75.56
Yes	285	24.44
Parathyroidectomy (*n*, %)		
No	982	84.22
Yes	184	15.78
Death (*n*, %)		
No	1023	87.74
Yes	143	12.26

*HD* hemodialysis

*P* value for categorical variables were estimated by* χ*² test or Fisher's exact test

*P* value for continuous variables were estimated by independent two samples

### Clinical features of the study population assessed by in-person visit

A total of 3509 in-person visits were analyzed: 812 had a low KPS score (< 80), 2697 a high KPS score (≥ 80). Patients with high KPS scores at in-person visits demonstrated significantly higher levels of hemoglobin (Hb), albumin, blood urea nitrogen (BUN), creatinine (Cr), phosphorus (P), sodium (Na), potassium (K), uric acid, aluminum, iPTH, Kt/V, nPCR, and higher ultrafiltration amounts at each HD session compared with patients with low KPS scores at in-person visits (Table 2). On the other hand, patients with high KPS scores at in-person visits demonstrated significantly lower leukocyte counts and lower Ca, glucose, alkaline phosphatase, ferritin, and CT ratios.

**Table 2:** Clinical features of the study population in-personvisit (*N* = 3509)

Variable	Karnofsky score				*P*
	Low (*N* = 812)		High (*N* = 2697)		
	Mean	SD	Mean	SD	
Hb (g/dL)	10.44	1.28	10.73	1.22	< 0.001
Hct (%)	32.16	4.03	32.87	3.78	< 0.001
WBC (10^3^/μL)	6.28	1.90	6.11	1.70	0.014
Albumin (g/dL)	3.69	0.31	3.92	0.29	< 0.001
BUN (mg/dL)	67.82	19.52	72.60	17.06	< 0.001
Creatinine (mg/dL)	9.46	2.23	11.36	2.28	< 0.001
Ca (mg/dL)	9.46	0.85	9.38	0.85	0.019
P (mg/dL)	4.78	1.45	5.11	1.43	< 0.001
Na (mEq/L)	135.60	3.62	136.74	3.19	< 0.001
K (mEq/L)	4.79	0.78	5.05	0.69	< 0.001
Glucose (mg/dL)	164.26	62.92	131.15	52.04	< 0.001
AST (IU/L)	19.41	7.21	19.00	7.05	0.149
Alkaline P (IU/L)	87.13	37.28	79.26	33.28	< 0.001
Cholesterol (mg/dL)	172.13	37.97	174.69	37.29	0.088
Triglyceride (mg/dL)	153.68	91.33	150.11	91.50	0.329
Uric acid (mg/dL)	7.14	1.43	7.54	1.42	< 0.001
Ferritin (ng/mL)	491.65	293.49	409.03	254.75	< 0.001
Al (μμg/dL)	1.12	0.84	1.23	0.90	0.001
iPTH (pg/mL)	295.98	340.86	338.58	343.49	0.002
Kt/V	1.63	0.28	1.68	0.31	< 0.001
nPCR	1.11	0.30	1.18	0.26	< 0.001
Ultrafiltration amount in each HD (L)	1.92	1.21	2.23	1.29	< 0.001
Cardiothoracic ratio	0.52	0.07	0.49	0.07	< 0.001

*P* value for continuous variables were estimated by independent two samples

Karnofsky score: low < 80; high ≥ 80

Ferritin, Al, iPTH, Kt/V, nPCR, Cardiothoracic ratio expressed by medium values, others expressed by mean values

*Hb* hemoglobin, *Hct* hematocrit, *Ca* calcium, *P* phosphorus, *Na* sodium, *K* potassium, *AST* aspartate aminotransferase, *Alkaline P* alkaline phosphatase, *Al* aluminum, *iPTH* intact parathyroid hormone, *nPCR* normalized protein catabolic rate, *HD* hemodialysis

### CART for predicting functional status measured by KPS scores

**Fig. 1:**
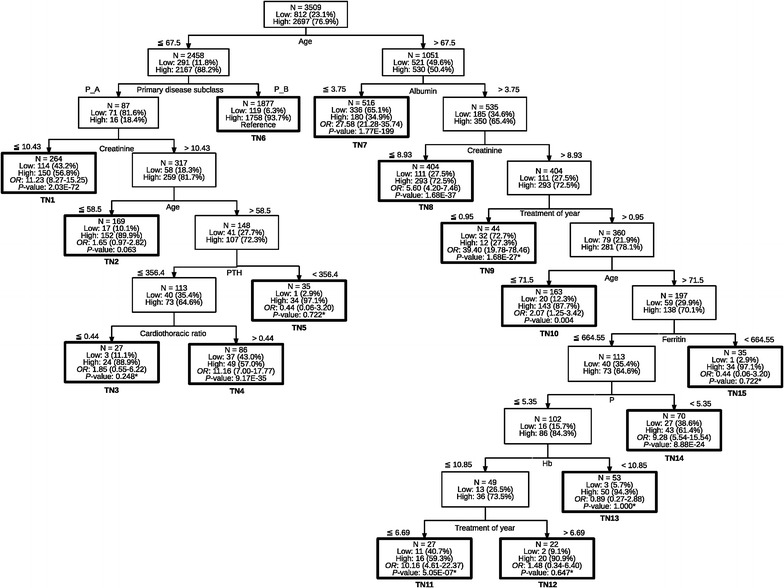
Classification tree for predicting Karnofsky Performance Status Scale in hemodialysis patients. TN: terminal node; *P value: *P* value for Fisher Chi Square test; *P* value: *P* value for Chi Square test; P_A, P_B items: see Additional file 1: Table S1

Figure 1 shows a “pruned” classification tree model for predicting functional status measured by KPS scores. The first variable chosen by the CART algorithm to split the data was age. Age ≤ 67.5 years was assigned to the left node, while age > 67.5 years was assigned to the right node. There were ten left nodes and five subgroups in the age ≤ 67.5 years node. In contrast, there were sixteen right nodes and eight subgroups in the age > 67.5 years node. In the age ≤ 67.5 years classification, the second variable chosen by the CART algorithm was the primary kidney disease subclass (Additional file 1: Table S1). The final variable chosen was the CT ratio. In the age > 67.5 years classification, the second variable chosen by the CART algorithm was albumin. The final variable chosen was treatment in years.

### Logistic regression analyses

Table 3 presents the logistic regression analysis results without interactions to predict the KPS score level at each in-person visit. Older age, higher Hct, BUN, glucose, and ferritin levels significantly increased the log-odds of obtaining a low KPS score level at in-person visits. Primary kidney disease subclass in P_B (Additional file 1: Table S1), the use of vitamin D, anti-hypertensive agents, longer treatment years, a higher number of HD sessions per week, longer HD duration, larger dialyzer surface area, higher Hb, albumin, creatinine, Na, K, aspartate aminotransferase (AST) levels, and a higher Kt/V value significantly decreased the log-odds of measuring a low KPS score during an in-person visit.

**Table 3:** Predicting Karnofsky score level at each in-person visit using the logistic regression model without interactions

Variable	Estimate	SE	*P* value
Intercept	13.19	2.79	< 0.001
Age	0.08	0.01	< 0.001
Gender (female as Ref.)	0.21	0.14	0.124
DM	− 0.05	0.32	0.875
Primary disease subclass (P_A as Ref.)	− 0.70	0.31	0.022
Use of vitamin D	− 0.27	0.13	0.045
Anti-hypertensive agent	− 0.40	0.11	< 0.001
Use of iron	− 0.14	0.17	0.417
Parathyroidectomy	− 0.02	0.17	0.910
Treatment of year	− 0.05	0.01	< 0.001
The number of times per week	− 0.57	0.18	0.002
Each dialysis time	− 0.56	0.21	0.007
Dialyzer surface area	− 0.95	0.22	< 0.001
Hb	− 0.93	0.18	< 0.001
Hct	0.33	0.06	< 0.001
WBC	− 0.01	0.03	0.837
Albumin	− 1.25	0.19	< 0.001
BUN	0.01	4.27E−03	0.026
Creatinine	− 0.21	0.03	< 0.001
Ca	0.03	0.07	0.669
P	0.07	0.04	0.125
Na	− 0.05	0.02	0.004
K	− 0.42	0.08	< 0.001
Glucose	2.48E−03	9.59E−04	0.010
AST	− 0.02	0.01	0.013
Alkaline P	2.92E−03	1.65E−03	0.078
Cholesterol	1.76E−03	1.46E−03	0.226
Triglyceride	4.41E−04	6.54E−04	0.500
Uric acid	− 0.05	0.04	0.281
Ferritin	4.05E−04	2.07E−04	0.050
Al	− 0.05	0.06	0.437
iPTH	2.10E−04	1.97E−04	0.287
Kt/V	− 0.70	0.22	0.001
nPCR	0.24	0.24	0.327
Ultrafiltration dehydration amount	0.07	0.04	0.118
Cardiothoracic ratio	1.08	0.80	0.178

Table 4 shows the results of predicting the KPS score level measured at each in-person visit using the logistic regression model including selected interactions. Older age, higher Hct, BUN, and glucose levels will significantly increase the log-odds of obtaining a low KPS score level. However, primary kidney disease subclass in P_B (Additional file 1: Table S1), the use of anti-hypertensive agents, a higher number of HD sessions per week, longer dialysis duration, larger dialyzer surface area, higher Hb, Na, K, AST levels, and a higher Kt/V value significantly decreased the log-odds of measuring a low KPS score level. For interaction results, the combination of older age with higher albumin level or longer treatment of years, and higher creatinine level with longer treatment years significantly decreased the log-odds of a low KPS score level, whereas for patients with the combination of primary kidney disease subclass (P_B) (Additional file 1: Table S1) and older age, and primary kidney disease subclass (P_B) and higher creatinine level, higher Hb levels and longer treatment years significantly increased the log-odds of having a low KPS score assessment.

**Table 4:** Predicting Karnofsky score level at each in-person visit using the logistic regression model including selected interaction

Variable	Estimate	SE	*P* value
Intercept	2.54	6.89	0.712
Age	0.20	0.07	0.007
Gender (female as Ref.)	0.25	0.14	0.075
DM	0.09	0.32	0.776
Primary disease subclass (P_A as Ref.)	− 6.18	1.12	< 0.001
Use of vitamin D	− 0.24	0.14	0.078
Anti-hypertensive agent	− 0.35	0.11	0.002
Use of iron	− 0.17	0.17	0.324
Parathyroidectomy	0.05	0.18	0.791
Treatment of year	− 0.02	0.12	0.876
The number of times per week	− 0.52	0.18	0.005
Each dialysis time	− 0.54	0.21	0.010
Dialyzer surface area	− 0.93	0.23	< 0.001
Hb	− 0.96	0.24	< 0.001
Hct	0.32	0.06	< 0.001
WBC	− 0.01	0.03	0.691
Albumin	2.14	1.62	0.188
BUN	0.01	4.32E−03	0.039
Creatinine	− 0.01	0.38	0.972
Ca	0.04	0.07	0.590
P	0.27	0.35	0.438
Na	− 0.04	0.02	0.009
K	− 0.42	0.08	< 0.001
Glucose	2.29E−03	9.69E−04	0.018
AST	− 0.02	0.01	0.040
Alkaline P	2.26E−03	1.70E−03	0.183
Cholesterol	1.07E−03	1.48E−03	0.470
Triglyceride	5.89E−04	6.61E−04	0.373
Uric acid	− 0.05	0.04	0.279
Ferritin	8.62E−04	1.64E−03	0.600
Al	− 0.02	0.06	0.704
iPTH	− 8.04E−04	1.46E−03	0.581
Kt/V	− 0.75	0.23	0.001
nPCR	0.29	0.24	0.241
Ultrafiltration dehydration amount	0.08	0.05	0.093
Cardiothoracic ratio	− 0.10	1.07	0.923
Age × albumin	− 0.04	0.02	0.043
Primary disease subclass × age	0.06	0.01	< 0.001
Primary disease subclass × creatinine	0.15	0.05	0.004
Albumin × creatinine	− 0.07	0.08	0.342
Creatinine × age	1.41E−03	2.85E−03	0.620
Creatinine × treatment of year	− 0.02	0.01	0.004
Age × iPTH	− 1.18E−05	1.63E−05	0.470
iPTH × cardiothoracic ratio	3.53E−03	2.29E−03	0.123
Treatment of year × age	− 2.36E−03	1.15E−03	0.041
Age × ferritin	− 1.23E−05	2.07E−05	0.553
Ferritin × P	7.21E−05	1.38E−04	0.602
P × Hb	− 0.02	0.03	0.434
Hb × treatment of year	0.03	0.01	0.001

## Discussion

In the present study, we used the CART algorithm and a logistic regression model to identify and predict the risk factors correlating with functional status during the maintenance of HD patients. The CART algorithm identified at-risk subgroups of functional impairment based on variable clinical parameters. The CART analysis results showed that the pattern of risk factors differed across the main node of age, and that outcomes were not related to dialysis adequacy, and were not strongly related to Hb levels. This raised an important issue regarding the relative importance of clinical variables versus variables that reflect individual factors, such as age or primary cause of renal disease. Nevertheless, the results did not reject the role of dialysis adequacy or treatment goal in clinical variables on the maintenance of functional status in HD patients. These results provide an alternative approach in the management of functional status in HD patients. The approach could include a rehabilitation program for the aged, continuous monitoring of primary renal disease, and prevention of relevant complications.

We also used logistic regression analysis as a complementary approach to identify risk factors for functional status in HD patients. Although the results illustrated that there was a slight difference between the two analyses, there was some overlap in the identification of risk factors. Variables such as age, primary renal disease, serum albumin and creatinine, treatment years stand as risk factors for functional impairment measured by KPS scores in HD patients. There is no definitive advantage or disadvantage when comparing classification tree analysis and logistic regression analyses. One approach may be better than the other in some situations. The advantages of CART analysis are (1) the flexibility to deal with numerous response types such as numerical data, categorical data, and ratings; (2) robustness of construction; (3) ease of interpretation; and (4) the ability to deal with missing values in response and explanatory variables. CART analysis complements many traditional statistical techniques, including logistic regression, loglinear models, and linear discriminant analysis [20]. Classification tree analysis captures sequential decision rules that may apply to subgroups of cohort based on variables having clinical utility or theoretical significance. In general, a regression analysis relatively weighs pervasiveness, while a classification tree analysis weighs specificity [21]. Based on our results, we propose that both methods can be complementary to explore the relevant clinical situation in dialysis patients. Nevertheless, additional clinical studies are required to further validate these methods.

When we compared the different results obtained from CART and logistic regression analyses, age was the main variable being able to predict low KPS scores in both analyses. Older age is associated with higher odds of frailty in patients receiving HD [22]. One component of frailty is low physical activity. Low KPS scores generally indicate low physical activity; however, measurements of physical activity in patients receiving HD could be biased if based on self-reported function. A comparison between self-reported function and a performance-based definition was reported in a well-designed study [23]. The researchers found that self-reported function achieved only 72.5% overall accuracy. In the present study, KPS scores were measured by trained HD nurses in the hemodialysis room, thereby preventing bias due to self-reported function from individual patients receiving HD. A previous study in a HD cohort with a cutoff age of 65 years demonstrated that physical aspect was independently associated with age and KPS score after 1 year of HD initiation [24, 25]. The authors also found elderly HD patients lost fewer QoL measures compared with younger HD patients. From these results, the authors concluded that elderly HD patients could make adaptation to HD with less difficulty and overcome most of the physical limitations induced by aging or dialysis therapy. Accordingly, our CART analysis revealed the cutoff age to be 67.5 years as the first split node. Furthermore, age demonstrated a powerful predictor of low KPS score by logistic regression analysis. Therefore, we conclude that age is the most important risk factor for functional status in HD patients. Nevertheless, we did not investigate the direct relationship between functional status and the physical or mental aspect in the study. A further well-designed cohort study is warranted to explore these relationships.

In the present study, we also examined the interaction effects on risk for the measurement of KPS scores by clinical variables. Some combinations were identified, which significantly correlated to KPS scores in HD patients. Indeed, older age with higher albumin level or longer treatment years, and higher creatinine level with longer treatment years significantly decreased the log-odds of obtaining a low KPS score in-person visit. In contrast, primary kidney disease with older age or higher creatinine level and higher Hb level with longer treatment years significantly increased the log-odds of measuring low KPS scores at an in-person visit. We recognized that higher albumin and creatinine levels indicated a better nutritional status. Nutritional status may reduce morbidity in dialysis patients [26, 27]. Consequently, good functional status should be expected in HD patients. Similarity, longer HD treatment years indicate better adaptation and clinical condition in HD patients. It also may preserve good functional status in HD patients. However, the interaction study demonstrated longer HD treatment years and higher Hb level would significantly increase the log-odds of measuring low KPS scores. A similar trend was also demonstrated in CART analysis in the older age subgroup. It seemed that the benefits of HD treatment years would be averted when combined with higher Hb levels. A clear explanation cannot be drawn from the present study. Similar results were also found in primary kidney disease with older age and higher creatinine levels by logistic regression analysis. In the CART analysis at a younger age node, primary kidney disease, creatinine, and age were the leading parameters that were able to split the data. It appeared that results obtained by both approaches showed a parallel comparison in the younger age subclass. Overall, the clinical variables by logistic regression interaction analysis, i.e., age, albumin, creatinine levels, and HD treatment years, indicated a favorable functional status in HD patients, which were similar to the selected clinical variables resulting from the CART algorithm.

## Conclusions

We present two tools to investigate the clinical variables related to functional status measured by KPS in HD patients. The comparison of results yielded by the CART algorithm with those produced by a logistic regression analysis was used to obtain complementary evidence on important risk factors for functional status in HD patients. Although the weight of contribution to the functional status in each clinical variable was slightly different, the overall trend was very similar in the CART and logistic regression analyses. The results can be used as a reference for clinicians to determine therapeutic strategy for functional improvement in maintenance of HD patients.

## Additional file

**Additional file 1: Table S1.** The primary kidney disease subclass.

## Abbreviations

CART: classification and regression tree approach
HD: hemodialysis
KPS: Karnofsky Performance Status
ESRD: end-stage renal disease
QoL: quality of life
SF: Short Form
iPTH: intact parathyroid hormone
nPCR: normalized protein catabolic rate
CT ratio: cardiothoracic ratio
Ca: calcium
DM: diabetes mellitus
Hb: hemoglobin
BUN: blood urea nitrogen
Cr: creatinine
P: phosphorus
AST: aspartate aminotransferase
