# Supplementary material for: Impact of risk factors on functional status in maintenance hemodialysis patients
Source: Eur J Med Res. 2017 Dec 28;22:54. doi: 10.1186/s40001-017-0298-1 (PMC5745856; doi:10.1186/s40001-017-0298-1)
Supplement: Supplementary file 1 — Additional file 1: Table S1. The primary kidney disease subclass. [file 40001_2017_298_MOESM1_ESM.doc]

| **Table S1**  P_A subclass  A-01B-j Analgesic nephropathy  A-01B-k Other glomerulopathy  A-02A Rapid progressive glomerulonephritis (no biopsy proof)  B-03 Diabetes mellitus  B-08 Gouty nephropathy  B-12 Other kidney failure caused by metabolic diseases  C-06 Other etiology caused urinary obstruction  D-05 Other renal vascular disease  E-01 Polycystic kidney disease  E-05 Hereditary diseases caused kidney failure  P-B subclass  A-01A Chronic Glomerulonephritis (no biopsy proof) |
| --- |
| A-01B Chronic Glomerulonephritis (biopsy proof) |
| A-01B-c Membranous glomerulopathy |
| A-01B-e Mesangial proliferative glomerulopathy |
| A-01B-f Minimal change disease |
| A-03A Chronic interstitial nephritis (no biopsy proof) |
| A-04 Chronic pyelonephritis |
| A-05 Acute kidney injury (not recovered) |
| A-06 Other renal parenchymal diseases |
| B-01 Nephrosclerosis |
| B-02 Malignant hypertension |
| B-04 Systemic lupus erythematous |
| B-09 Liver cirrhosis |
| B-10 Heart failure |
| B-11 Eclampsia |
| B-13 Other kidney failure caused by systemic disease |
| C-01 Urinary tract stone |
| C-03 Urinary malignancy |
| C-04 Tumors caused urinary obstruction |
| D-04 Hemolytic uremic syndrome |
| F Other known causes of kidney failure |
| G Unknown cause of kidney failure |
